# Outlier Detection Using Improved Support Vector Data Description in Wireless Sensor Networks

**DOI:** 10.3390/s19214712

**Published:** 2019-10-30

**Authors:** Pei Shi, Guanghui Li, Yongming Yuan, Liang Kuang

**Affiliations:** 1 School of IoT Engineering, Jiangnan University, Wuxi 214122, China; ship@ffrc.cn; 2 Freshwater Fisheries Research Center of Chinese Academy of Fishery Sciences, Wuxi 214081, China; Yuan@ffrc.cn; 3 School of IoT Engineering, Jiangsu Vocational College of Information Technology, Wuxi 214153, China; kuangliang.89@163.com

**Keywords:** wireless sensor networks (WSNs), outlier detection, support vector domain description, Parzen-window algorithm, water quality monitoring

## Abstract

Wireless sensor networks (WSNs) are susceptible to faults in sensor data. Outlier detection is crucial for ensuring the quality of data analysis in WSNs. This paper proposes a novel improved support vector data description method (ID-SVDD) to effectively detect outliers of sensor data. ID-SVDD utilizes the density distribution of data to compensate SVDD. The Parzen-window algorithm is applied to calculate the relative density for each data point in a data set. Meanwhile, we use Mahalanobis distance (MD) to improve the Gaussian function in Parzen-window density estimation. Through combining new relative density weight with SVDD, this approach can efficiently map the data points from sparse space to high-density space. In order to assess the outlier detection performance, the ID-SVDD algorithm was implemented on several datasets. The experimental results demonstrated that ID-SVDD achieved high performance, and could be applied in real water quality monitoring.

## 1. Introduction

Wireless sensor networks (WSNs) have been widely used in various fields, such as industrial (e.g., industrial surveillance), military (e.g., military reconnaissance), medical (e.g., medical diagnosis), agricultural (e.g., agriculture production detection), mechanical engineering, and aerospace engineering applications [1,2,3,4,5,6,7,8]. The reliability of sensor data has increasingly attracted attention from both academia and industry. Outlier detection can recognize noise, errors, events, and hostile attacks, which helps to reduce network risk and ensure data quality [9,10,11,12]. Generally, outliers are less than the normal data in the monitoring process, and they can represent changes in monitoring objects and environments. Therefore, outliers have great potential value. In aquaculture, sensors are susceptible to germ corrosion and easily go wrong since they are deployed underwater [13]. Moreover, in the water quality monitoring process, a high speed of outlier detection is required for processing big data.

There are four commonly used outlier detection methods: statistical-based [14,15], nearest neighbor [16,17], clustering-based [18,19], classification-based [20,21,22], etc. However, these methods still have some limitations when they are used in practice. The statistical-based methods construct models based on prior knowledge [23]. There is no mathematical model to match the real application problem of WSNs perfectly. The nearest neighbor method is a classic detection algorithm [24], and it is time-consuming and has poor scalability when applied in high-dimensional data. Clustering-based methods are limited to the issue of clustering width [25]. Meanwhile, the calculation of data distance consumes computational resources in high-dimensional datasets. Therefore, this method is unsuitable for the limited-power devices used in WSN applications. Classification-based methods include Bayesian network theory, support vector machine, etc. Bayesian networks can obtain the correlations among data, but they have poor scalability for high-dimensional data. Although the calculation is complex, support vector machines (SVMs) have been introduced to outlier detection for their advantages in solving binary classification problems.

Support vector data description (SVDD) is a widely used one-class support vector machine (OC-SVM) proposed by Tax and Duin [26]. It is an unsupervised learning method suitable for detecting outliers in the fault monitoring process. Jae [27] applied SVDD to classify normal behavior patterns and to detect abnormal behavioral patterns. Bovolo [28] utilized change vector analysis (CVA) and SVDD for a change detection problem. Khediri [29] presented a procedure based on the kernel *k*-means clustering and SVDD to separate different nonlinear process modes and to effectively detect faults in the etch metal process. Liu [30] presented a high-speed inline defect inspection scheme based on fast SVDD for a thin-film transistor (TFT) array process of thin-film transistor liquid crystal display (TFT-LCD) manufacturing. Zhao [31] used an SVDD-based method in pattern-recognition-based chiller fault detection.

However, most outlier detection algorithms based on SVDD only take the kernel-based distance between the spherical boundary and the data point into account, ignoring the distribution of data. Therefore, many researchers have conducted experiments on SVDD to improve the fault detection performance [32,33]. Lee [32] proposed a distance measurement to SVDD based on the notion of a relative density degree for each data point in order to reflect the distribution of a given dataset. Cha [33] imported the notion of density weight to SVDD, which is the relative density of each data point based on the density distribution of the target data using the *k*-nearest neighbor (*k*-NN) approach. However, this approach requires more calculation. When used in an unbalanced dataset, the performance will be unstable.

In this paper, we develop a new method based on existing studies [32,33], namely, the improved density-compensated SVDD algorithm (ID-SVDD). The relative density weight is used to search for an optimal SVDD. In contrast to the existing studies, we obtain the relative density weight by the exponentially weighted Parzen-window density. We incorporate it with SVDD to help obtain the distribution of the target data. All data points are efficiently mapped from sparse space to high-density space. Then, the Mahalanobis distance is utilized to improve the Gaussian window function, which can eliminate the interference of correlations between variables. The traditional SVDD, density-weighted SVDD (DW-SVDD), and density-compensated SVDD (D-SVDD) are compared with ID-SVDD. Experimental results indicate that the detection accuracy and efficiency were both improved by ID-SVDD, and that it could be applied for outlier detection in real water quality monitoring processes.

The paper is organized as follows: in Section 2, we introduce the traditional SVDD method and the ID-SVDD method. In Section 3, we conduct experiments and demonstrate the effectiveness of ID-SVDD for outlier detection. In Section 4, we give conclusions for this study.

## 2. Methodology

### 2.1. Support Vector Data Description

**Figure 1:**
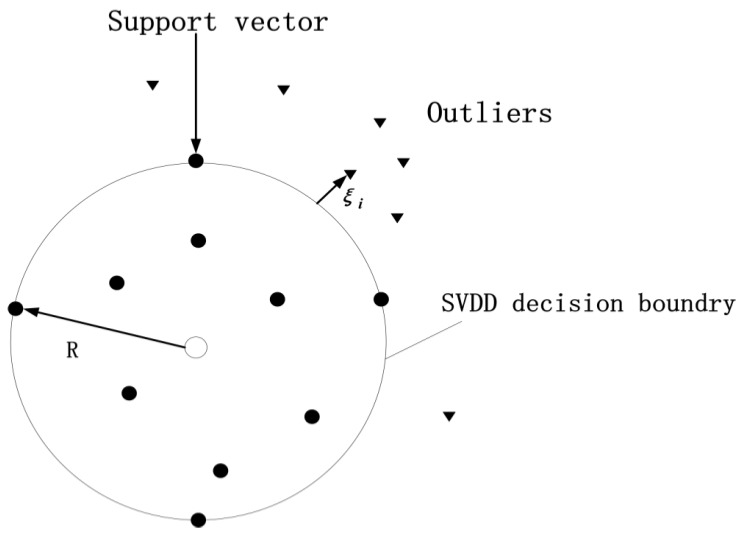
Illustration of support vector data description (SVDD) in feature space for outlier detection.

SVDD is a common data classification algorithm proposed by Sun and Tsung [34]. The basic idea is to map target data to high-dimensional feature space, and constructing a data description as the smallest sphere to contain all possible target data [35]. The objective of SVDD is to find a spherical boundary with minimal radius *R* with center o, and realize the classification of unknown data. The data points inside the sphere are the target class, and those outside are treated as the non-target class. For ease of reference, Table 1 summarizes the key notations. The details of SVDD are illustrated in Figure 1.

Given target data {*x_i_*, *i =* 1, 2, …, *n*}, SVDD maps the target data from input space into a feature space *F* via nonlinear mapping function φ and gets the smallest sphere Ω = (o,*R*) in *F*. The objective function of SVDD is as follows:

(1) $$\left\{ \begin{array}{l} \min\;F(R,a,\xi_{i})=R^{2}+C\sum_{i=1}^{n}\xi_{i}\\ s.t.\;{\left\|\varphi (x_{i})-a\right\|}^{2}\leq R^{2}+\xi_{i},\;\xi_{i}\geq 0,\;\forall i=1,2,\cdots ,n \end{array}\right.$$

where *C* is a parameter that denotes the trade-off between sphere volume and the number of target data outside the sphere. The slack variable *ξ_i_* is used to incorporate the effect of data not included in the data description, which allows a probability that some points can be wrongly classified.

To solve the objective function (1), we introduce the Lagrange multiplier *α.* By calculating the inner product with the kernel function, we can get the rearranged function as follows:

(2) $$\left\{ \begin{array}{l} \max\;\sum_{i=1}^{n}\alpha_{i}K(x_{i}\cdot x_{i})-\sum_{i=1}^{n}\sum_{j=1}^{n}\alpha_{i}\alpha_{j}K(x_{i}\cdot x_{j})\\ s.t.\;0\leq \alpha_{i}\leq C,\;i=1,2,\cdots ,n\\ \;\;\sum_{i=1}^{n}\alpha_{i}=1 \end{array}\right.$$

where *K*(*x_i_*,*x_j_*) is the kernel function that satisfies Mercer’s theorem [36]. The radius *R* of the sphere and the distance *r* between an observation datum in the feature space and center o are denoted as:

(3) $$R^{2}=K(z\cdot z)-2\sum_{i=1}^{n}\alpha_{i}K(z\cdot x_{i})+\sum_{i=1,j=1}^{n}\alpha_{i}\alpha_{j}K(x_{i}\cdot x_{j})$$

(4) $$r^{2}=K(x_{k}\cdot x_{k})-2\sum_{i=1}^{n}\alpha_{i}K(x_{i}\cdot x_{k})+\sum_{i=1,j=1}^{n}\alpha_{i}\alpha_{j}K(x_{i}\cdot x_{j})$$

In outlier detection, Figure 1 also shows the SVDD detection principle. It determines the description boundary as the detection boundary. For given test data, *x* is regarded as a target datum inside the sphere if *r* ≤ *R*, which indicates *x* is normal. Otherwise, it is treated as an outlier, which indicates *x* is abnormal.

### 2.2. Density-Compensated Support Vector Data Description

The traditional SVDD algorithm often ignores the impact of data density distribution on classification [33], which means the sphere cannot reflect all features of the target data, and reduces the classification accuracy. To account for the distribution information of data, we introduce the notion of relative density weight to compensate SVDD, which reflects how dense the region of target data is compared to other regions. This approach makes the training data in high-density areas more likely to fall into the sphere than those in low-density areas.

In this paper, the Parzen-window algorithm [37] is applied to calculate the relative density weight of sample data. Assuming the target data *X =* {*x_1_, x_2_, …x_i_, i =* 1, 2, …, *n*}, the relative density weight of point *x_i_* in a dataset is determined as:

(5) $$\rho_{i}=\exp\{\omega \times \frac{Par(x_{i})}{\theta }\},\forall i=1,2,\cdots ,n$$

(6) $$Par(x_{i})=\frac{1}{n}\sum_{j=1}^{n}\left(\frac{1}{\sqrt{{\left(2\pi \right)}^{d_{S}}}})\exp(-\frac{1}{2s}{\left\|x_{i}-x_{j}\right\|}^{2}\right)$$

where *ρ_i_* is the relative density weight, $\theta =\frac{1}{n}\sum_{i=1}^{n}Par(x_{i})$ is the mean Parzen-window density *Par*(*x_i_*)*, d* represents the feature dimension of input data, *ω* (0 ≤ *ω* ≤ 1) is the weighting factor, *s* denotes the smoothing parameter of Parzen-window density, and *n* is the number of target data.

We use relative density to reflect the data distribution in real space. In the process of searching for an appropriate description, we calculate the relative density weight according to Equation (5). After importing the relative density weight to SVDD, we obtain the redefined objective function as follows:

(7) $$\left\{ \begin{array}{l} \min\;F(R,a,\xi_{i})=R^{2}+C\rho (x_{i})\sum_{i=1}^{n}\xi_{i}\\ s.t.\;{\left\|\varphi (x_{i})-a\right\|}^{2}\leq R^{2}+\xi_{i},\;\xi_{i}\geq 0,\;\forall i=1,2,\cdots ,n \end{array}\right.$$

Let the relative density weight multiply the slack variable. Then each datum in high-density regions will get a high relative density value. For searching the optimal description of target data, D-SVDD can shift the description boundary to the dense areas. By introducing the Lagrange multiplier to solve Equation (7) in SVDD, we get the optimization Equation (8):

(8) $$\left\{ \begin{array}{l} \max\;\sum_{i=1}^{n}\alpha_{i}K(x_{i}\cdot x_{i})-\sum_{i=1}^{n}\sum_{j=1}^{n}\alpha_{i}\alpha_{j}K(x_{i}\cdot x_{j})\\ s.t.\;0\leq \alpha_{i}\leq \rho (x_{i})C,\;i=1,2,\ldots ,n\;\\ \;\;\sum_{i=1}^{n}\alpha_{i}=1 \end{array}\right.$$

### 2.3. Outlier Detection Using Improved Density-Compensated SVDD

In D-SVDD, we choose the Gaussian function as the window function of the Parzen-window algorithm and use the Euclidean distance to measure the distance in the Gaussian function. However, Euclidean distance does not take into account the correlation between sample points [38], which will affect the precision of D-SVDD. Mahalanobis distance (MD) is scale-invariant [39], which can overcome the shortcomings of Euclidean distance. Thus, it can avoid the calculation error caused by measurement units or the difference in magnitude of eigenvector values [40]. The performance of MD is better than Euclidean distance. MD is a non-uniform distribution of the normalized distance in Euclidean space, and it is constant for all linear transformations. The formula of MD is given as follows:

(9) $$md_{ij}=\sqrt{{\left(x_{i}-x_{j}\right)}^{T}S^{-1}(x_{i}-x_{j})}$$

(10) $$MS=\frac{1}{n-1}\sum_{i=1}^{n}\left(x_{i}-\bar{x}\right){\left(x_{i}-\bar{x}\right)}^{T}$$

where *md_ij_* is the Mahalanobis distance between vector *x_i_* and *x_j_*, *MS* is the covariance matrix between two vectors, and $\bar{x}=\frac{1}{n}\sum_{i=1}^{n}x_{i}$ denotes the mean of *x_i_*.

In this paper, we introduce the MD to replace Euclidean distance in the Gaussian function. By calculating the MD between data points, the improved Parzen-window density for *x*_1_ is redefined. The Parzen-window relative density weight is denoted as

(11) $$IPar(x_{i})=\frac{1}{n}\sum_{j=1}^{n}\left(\frac{1}{\sqrt{{\left(2\pi \right)}^{d_{S}}}})\exp(-\frac{1}{2s}md_{ij}^{2}\right)$$

(12) $$\rho_{i}=\exp\{\omega \times \frac{IPar(x_{i})}{\theta }\},\forall i=1,2,\cdots ,n$$

Now, we can give the pseudocode of the ID-SVDD algorithm as shown in Algorithm 1. The outputs of the ID-SVDD algorithm are the Lagrange multipliers *α_i_*, radius *R* of sphere, and distance *r*. For a given *x_i_*, it is classified as an outlier if the distance *r_i_* is greater than *R*. If not, *x_i_* is classified as a normal datum.

**Algorithm 1.** ID-SVDD outlier detection
**Input:**
Target dataset *X =* {*x_1_*, *x_2_*, …*x_i_, i* = 1, 2, …, *n*}, kernel function *K*(.)
**Output:**
α_i_, *R*, and *r*.
**Begin**
Define an array *P* to store relative density weight for each point.for (*k* = 1; *k* ≤ *n*; *k*++) docalculate *P_k_* = *ρ*(*x_k_*) according to Equation (12)EndSolve the optimization problem of (8).Determine a sample whose *α_i_* is between 0 and *ρ*(*x_i_*)*C*.Calculate the radius *R* of sphere and the distance *r* according to Equations (3) and (4).
**End**
**Return***α_i_*, *R* and *r*.

## 3. Experiments

### 3.1. Experiment Design

In order to evaluate the performance of ID-SVDD, we compared it with the traditional SVDD, D-SVDD, and DW-SVDD provided in [33]. Cha proposed the DW-SVDD with a weight coefficient calculated by *k*-NN distance. We chose DW-SVDD for comparison because it also attempts to apply the relative density to traditional SVDD. Meanwhile, these four methods were implemented with MATLAB language and run on a PC with 2.9-GHz Core™ processor, 16.0 G memory, and Microsoft Windows 10 operating system.

Considering the completeness and continuity of data, we chose the data of nodes 12 and 17 in the SensorScope system dataset [41] to complete simulation experiments. The SensorScope system is deployed at Grand-St-Bernard Mountain, which lies between Switzerland and Italy. The datasets have two attributes, including the external temperature and surface temperature. In addition, we finished experiments on a real water quality dataset with three attributes, including dissolved oxygen (DO), pH, and dissolved oxygen relative saturation. More detailed information about the experimental datasets is presented in Table 2.

We used different indexes to evaluate the performance of ID-SVDD. These indexes included true positive rate (TPR), true negative rate (TNR), accuracy, and run time [42,43]. The calculation formulas of these indexes are shown as follows.

(13) $$TPR=\frac{TP}{TP+FN}$$

(14) $$TNR=\frac{TN}{FP+TN}$$

(15) $$\mathrm{Accuracy}=\frac{TP+TN}{\left(TP+FN)+(FP+TN\right)}$$

where *TP* is the number of true positive results, *TN* represents the number of true negative results, *FP* is the number of false positive results, *FN* denotes the number of false negative results. These indicators together with run time can measure the performance of outlier detection methods effectively.

### 3.2. Experiment Results

#### 3.2.1. Comparison among Different Kernel Functions

The kernel function of SVDD can map nonlinear relations to higher-dimensional space and construct linear regression for processing [44]. In ID-SVDD, the kernel function also plays a key role. The common kernel functions include the linear kernel function (16), polynomial kernel function (17), Gaussian kernel function (18), and Sigmoid kernel function (19) [45]:

(16) k(x,z)=x⋅z

(17) $$k(x,z)={\left((x\cdot z)+1\right)}^{m}$$

(18) $$k(x,z)=\exp\left\{-\frac{||x-z|{|}^{2}}{2\delta^{2}}\right\}$$

(19) k(x,z)=tanh(k(x,z)+e), (k>0,e<0)

Here, we set the parameter *C* within the range 2^−8^ to 2^8^, which controls the trade-off between volume and errors. For better outlier detection results, we conducted experiments to choose the optimal kernel function. For the variable *δ* in Gaussian kernel function, we set it between 2^−8^ and 2^8^. Further, we used fivefold cross validation (CV) to find the adequate parameters of these kernel functions. After parameter selection, we obtained the optimal results of ID-SVDD with the SensorScope dataset. The detailed results are provided in Table 3.

Table 3 clearly indicates that the TPR, TNR, and accuracy of Gaussian kernel function were superior to the other three kernel functions in nodes 12 and 17 of SensorScope. Based on these experimental results, we adopted the Gaussian function as the kernel function of ID-SVDD in the outlier detection of water quality data.

**Figure 2:**
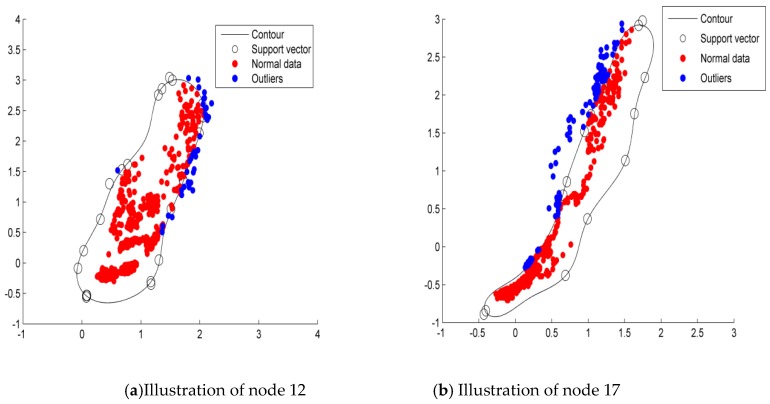
Distribution of the SensorScope dataset.

Figure 2a,b are the testing results distribution diagrams of the ID-SVDD detection algorithm in SensorScope node 12 and 17 datasets, respectively. From Figure 2, the support vector constructed the boundary to distinguish the normal data from outliers. The decision boundaries of the two datasets are both irregular graphics. The blue points outside the sphere represent the outliers, whereas the red points inside the sphere are normal data. It is clear that the detection model could describe the data edges accurately. So, the ID-SVDD model is an effective detection model.

#### 3.2.2. Comparison Results of Different Datasets

We conducted experiments to compare ID-SVDD with traditional SVDD, D-SVDD, and DW-SVDD in a set of standard datasets from SensorScope. The detection results are displayed in Table 4.

We can see from Table 4 that the TPR and accuracy values of ID-SVDD in nodes 12 and 17 were both superior to the D-SVDD, DW-SVDD, and SVDD. However, the TNR of ID-SVDD was lower than the TNR for D-SVDD and SVDD. These results indicate that the MD improved Parzen-window relative density weight could eliminate the interference of correlation between variables. It is appropriate for measuring the distance between target data. Meanwhile, the TPR*,* TNR, and accuracy of D-SVDD in nodes 12 and 17 were superior to those for DW-SVDD and SVDD. These results indicate that Parzen-window relative density weight is appropriate for compensating the SVDD. In terms of run time, these four algorithms were close. Actually, the use of ID-SVDD provided an acceptable improvement in outlier detection on the SensorScope datasets of nodes 12 and 17.

#### 3.2.3. Experimental Results on Water Quality Datasets

This experiment evaluated the ID-SVDD algorithm on a real water quality dataset. All data were collected from the internet of things (IOT) monitoring system running in the Nanquan breeding base located in Wuxi city, Jiangsu province [46]. This system uses various types of sensors to collect water quality data (e.g., DO, pH, and dissolved oxygen relative saturation). These data were transmitted from sensors to a server via the IOT monitoring system.

**Figure 3:**
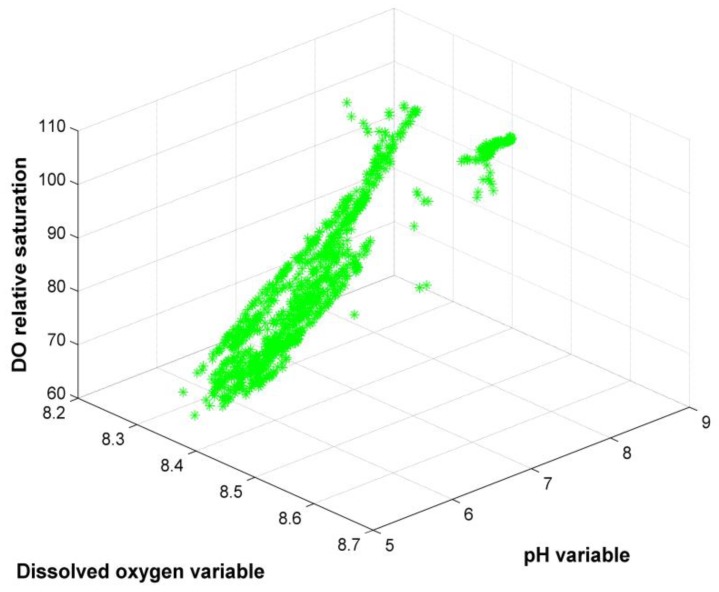
Illustration of the water quality dataset distribution in the training process. DO: dissolved oxygen.

The water quality dataset in this experiment included 1756 data (sampled 10 min once) from 20 May to 2 June 2017. We chose the first 1052 data as a training dataset, and the remaining 704 data as a testing dataset. The distribution of training data is illustrated in Figure 3.

**Figure 4:**
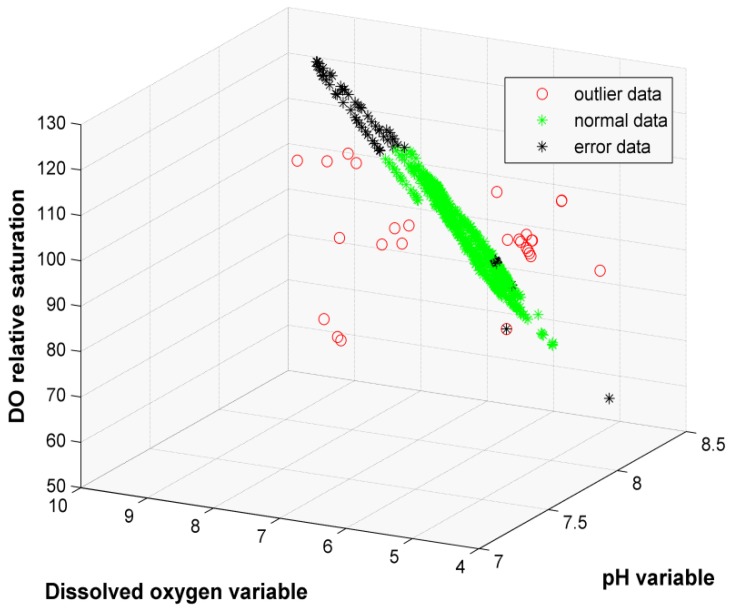
Detection results of the water quality dataset in the testing process.

Figure 3 is the training result distribution diagram of the ID-SVDD detection algorithm in the water quality dataset. In Figure 3, the green points represent all normal data in the training process. The three-dimensional coordinate represents the DO content, pH variable, and DO relative saturation variable, respectively. Most normal data are aggregated and distributed in an irregular shape. Small amounts of data are dispersedly distributed. The detection result on the testing dataset is shown in Figure 4.

**Figure 5:**
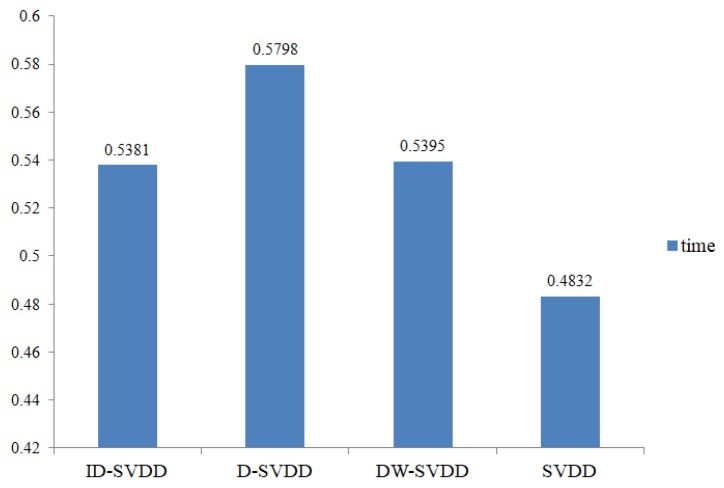
Outlier detection time of the water quality dataset with different algorithms.

We can see from Figure 4 that the three-dimensional coordinate is the same as Figure 3. After ID-SVDD outlier detection, the error points are shown in Figure 4 with the form of a black dot. Error points appear in both the normal dataset and the outlier dataset. The outlier data are distributed around the normal data. To evaluate the performance of the ID-SVDD algorithm, we made a comparison with D-SVDD, DW-SVDD, and traditional SVDD. The precision comparison results are shown in Table 5. Figure 5 presents the run-time comparison of the four algorithms.

It can be seen from Table 5 that ID-SVDD had the highest values of TPR and TNR, with 91.335% detection accuracy. The TPR of ID-SVDD was 2.322%, 28.542%, and 35.307% higher than those of D-SVDD, DW-SVDD, and traditional SVDD, respectively. The TNR of ID-SVDD was 4% greater than that of DW-SVDD, and equal to that of SVDD. ID-SVDD was successful in detecting the outliers of water quality data, increasing the accuracy by 2.064%, 27.327%, and 33.403% when compared to D-SVDD, DW-SVDD, and SVDD, respectively. There are correlations among pH, DO, and DO relative saturation. MD improved Parzen-window relative density weight can eliminate the interference of correlations, thus improving the detection performance. Meanwhile, the TPR*,* TNR, and accuracy of D-SVDD were superior to DW-SVDD and SVDD. That is because Parzen-window relative density weight can obtain a characterized description of the dataset in high-dimensional feature space and help search for an optimal SVDD. This approach is suitable for calculating the relative density weight. The introduction of improved relative density to SVDD helps enhance the performance for outlier detection efficiently.

As Figure 5 indicates, ID-SVDD had an advantage over D-SVDD and DW-SVDD in terms of run time. It consumed 0.5381 s for outlier detection. Single SVDD provided the shortest time (0.4832 s), but its TPR and accuracy were the lowest among the four algorithms. Therefore, ID-SVDD provided satisfactory outlier detection accuracy and efficiency, and it is suitable for detecting outliers in real water quality monitoring.

## 4. Conclusions

This paper presents a new outlier detection algorithm (ID-SVDD) incorporating the relative density weight with SVDD. This approach can obtain the features of the data, thus improving the performance of SVDD. To measure the relative density weight, we used the Parzen-window method. The Mahalanobis distance was applied to improve the Gaussian function in the calculation of relative density. ID-SVDD can realize data mapping from relatively sparse space to high-density space. We conducted experiments to evaluate the performance of ID-SVDD based on SensorScope datasets and water quality datasets, and then compared it with D-SVDD, DW-SVDD, and SVDD algorithms. The experimental results showed that ID-SVDD performed better than its three other counterparts in terms of TPR, TNR, accuracy, and run time. Therefore, it is efficient and useful to introduce relative density to SVDD. ID-SVDD provides a new idea of outlier detection and it can be used in real-world applications.

## Figures and Tables

**Table 1:** Key notations.

Symbol	Description
*R*	Radius of sphere
o	Center of sphere
*C*	The trade-off between sphere volume and the number of target data outside the sphere
*ξ_i_*	Slack variable
*α*	Lagrange multiplier
*R*	The distance between an observation datum in the feature space and center *a*
*θ*	The mean of Parzen-window density Par(*x_i_*)
*d*	The feature dimension of input data
*w*	Weighting factor
*n*	The number of target data
*ρ_i_*	Relative density weight of *x_i_*
*Par(x_i_)*	Parzen-window density of *x_i_*
*md_ij_*	Mahalanobis distance between vectors
*MS*	Covariance matrix
$\bar{x}$	Mean value of *x_i_*
*P*	Relative density weight array
*TP*	The number of true positive results
*TN*	The number of true negative results
*FP*	The number of false positive results
*FN*	The number of false negative results
*m*	The degree of polynomia
*δ*	Bandwidth of Gaussian kernel function
*k*	A constant
*e*	A constant

**Table 2:** Experimental datasets.

Datasets	Attributes	Normal Data	Outliers
SensorScope node12	2	1411	44
SensorScope node17	2	1309	137
water quality data	3	1706	50

**Table 3:** Comparison among different kernel functions. TNR: true negative rate; TPR: true positive rate.

Different Kernel Functions	TPR (%)	TNR (%)	Accuracy (%)
SensorScope12			
Linear	89.3525	0	84.4898
Ploy	100	0	94.5578
Gaussian	99.4245	87.5	98.7755
Tanh	98.1295	20	93.8776
SensorScope17			
Linear	38.4095	28.1482	36.5014
Ploy	92.5550	45.9259	83.8843
Gaussian	100	97.037	99.449
Tanh	68.1895	0	55.5096

**Table 4:** Detection results of SensorScope datasets.

	ID-SVDD	D-SVDD	DW-SVDD	SVDD
Node 12				
TPR (%)	99.4245	98.4173	70.5036	98.0496
TNR (%)	87.5	100	82.5	100
Accuracy (%)	98.7755	98.5034	71.1565	98.1788
Time (s)	0.489	0.5329	0.4211	0.5463
Node 17				
TPR (%)	100	98.8338	90.3553	99.3232
TNR (%)	97.037	100	63.7037	98.5185
Accuracy (%)	99.449	98.8981	85.3994	99.1736
Time (s)	0.3794	0.578	0.4172	0.3763

**Table 5:** Detection results for the water quality dataset. D-SVDD: density-compensated SVDD; DW-SVDD: density-weighted SVDD; ID-SVDD: improved density-compensated SVDD.

pond13	ID-SVDD	D-SVDD	DW-SVDD	SVDD
TPR (%)	91.1374	89.0694	70.901	67.356
TNR (%)	96.2963	100	92.5926	96.2963
Accuracy (%)	91.3352	89.4886	71.733	68.4659
